# Case of Severe Luxation of the Jaw Reduced

**Published:** 1814-03

**Authors:** A. Kington


*For the Medical and Physical Journal.*

### *Case of severe Luxation of the Jaw reduced; by* Mr. A. Kington.

On Christmas-day last, I was requested to visit Thomas Vaughn, a strong muscular man, who was frequently the subject of a dislocated jaw. Upon my arrival, I found his jaw had been luxated three days, and very violent efforts had been made use of to reduce it by an ignorant farrier, who resided in the house with him. The right side of his face was much swollen, and very painful. I endeavored to reduce the bone by placing my thumbs upon the molares, but had not sufficient strength to do it. I then had recourse to the method recommended by Mr. Fox, but finding the muscles so strongly in action, I thought proper to desist. I ordered him a strong cathartic, composed of Sulph. Magnes. ℥j. Infus. Senna ℥ij. to be taken immediately; to foment the part affected, and make use of the warm bath, until I saw him the next day.

26th.—The side of the face is more painful than yesterday; the patient very irritable, and would scarcely allow me to examine the parts. The muscles about the mouth are continually in action, and the mouth is drawn in different directions. The tongue appears to partake of the same affection as the muscles of the mouth, and he speaks with great difficulty. The cathartic I ordered yesterday not operating sufficiently, I directed it to be repeated every three hours, until copious evacuations were procured; and applied an emollient cataplasm to the swollen surface. His pulse being full and quick, I took away twelve ounces of blood from his arm.

27th.—I found his symptoms much exasperated, with the muscles of the mouth more affected by spasm than yesterday. His countenance exhibiting a ghastly appearance, I was of opinion this spasmodic action proceeded from the violence made use of by the farrier, and thought the only means of saving my patient was to remove the cause of irritation, by reducing the luxated bone as soon as possible. With this view I took away eighteen ounces of blood from his arm, ordered the parts surrounding the jaw to be continually fomented, and gave him a nauseating dose of Ant. Tart. every four hours.

28th.—I found my patient much debilitated, the swelling a little subsided, and the spasmodic affection of the muscles of the mouth somewhat abated. I now placed my thumbs upon the molares, made a slight degree of pressure in the usual way, and, to my great astonishment, the jaw slipt into its place immediately. From this time my patient gradually recovered, and was perfectly well at the end of a week, except a little spasm, which sometimes affected the levator anguli oris muscle, but entirely left him in the course of a month. He now continues in good health, and is able to masticate hard substances.

*Hereford, Feb.* 8, 1814.

